# 

**Published:** 1913-01-18

**Authors:** 


### Answers to Correspondents.

A. F. S., Dulwich.—The payment of 4s. 6d. a week, with simple lunch each day, for services rendered on six half-days a week would appear to be remuneration at a rate somewhere between 1s. 6d. and 2s. a day as calculated by the rules furnished by the Commissioners. If the young woman employed is over twenty-one years of age you will have to affix a 5d. stamp to her card each week, and you will be entitled to deduct 1d. from the weekly wage. If, however, the young woman is under twenty-one years of age, the full contribution of 6d. a week is payable, and you will be entitled to deduct 3d. a week from her wage. Marriage makes no difference to the contribution payable.

### Buildings Contemplated and in Progress.

Arbroath.—£12,000 is the sum authorised to be expended on the reconstruction of Arbroath Infirmary, and the directors are considering the ways and means of raising the total amount required—£14,500.

Barnet.—The medical officer has reported to the Barnet Guardians that in his opinion a block of three storeys could be added to the present infirmary at an approximate cost of £5,000. The block would accommodate 90 to 100 additional beds, operating theatre, dispensary, medical officer's room, etc. The committee recommend that a plan should be prepared, and the Guardians instructed Mr. Waymouth to make a rough sketch scheme at a cost not exceeding ten guineas.

Billericay (Essex).—Mr. Hugo R. Bird, St. Thomas Gate, Brentwood, is architect for alterations and additions at the isolation hospital. The works include the erection of a pavilion, isolation block, and laundry block. Tenders are invited.

Bolton.—The Guardians have adopted a scheme for extending the cottage homes at an estimated cost of £17,974.

Chester.—New buildings are projected at Upton Asylum, Chester, including isolation block, mortuary, workshops, and cottages. Mr. H. Beswick, F.R.I.B.A., of Newgate Street, Chester, is the architect.

Douglas.—At a cost of about £23,000 the new Noble's Hospital, situate in Westmoreland Road, has been built and is now formally opened.

Easington.—The Rural District Council has agreed to apply for sanction to a loan of £13,000 for building and furnishing the proposed isolation hospital.

Forest of Dean.—Mr. J. C. Tregellis, of 126 Bishopsgate, E.C., is architect for a memorial hospital at Forest of Dean.

Glasgow.—Alterations and additions are proposed at Glasgow Royal Infirmary. The plans have been approved by the Dean of Guild Court.

Gloucester.—The Guardians have confirmed their decision to build a new infirmary on Budding's Field at a cost not exceeding £28,200.

Great Yarmouth.—Messrs. Olley and Haward, Queen Street, Great Yarmouth, are architects for receiving home and hospital at Gorlestone and for installation of electric light throughout all the premises. Tenders are invited for the works.

Hamilton.—The Town Council has decided to build a new hospital at an estimated cost of £20,000.

Holywell.—Eleven tenders have been received by Holywell Guardians (North Wales) for the erection of a new infirmary from plans, etc., prepared by Messrs. J. H. Davies and Sons, of 14 Newgate Street, Chester. The lowest amounts to £6,735, the highest to £8,378.

Hornsey.—Tenders are invited by the Town Council for the supply and erection of laundry machinery, boiler, and disinfector at the Borough Isolation Hospital, Muswell Hill. Particulars may be obtained from Mr. E. J. Lovegrove, Borough Engineer, Municipal Offices, Highgate, N.

Manchester.—The Manchester Guardians propose to make alterations at Crumpsall Workhouse from particulars prepared by Mr. A. J. Murgatroyd, architect, 23 Strutt Street, Manchester. Tenders are being considered.

Norwich.—Messrs. Morgan and Buckingham, Upper King Street, Norwich, are architects for a block of children's homes at the Guardians' premises. Of ten tenders received the highest is £3,032, and the lowest £2,365.

Sheffield.—In November of last year the Ecclesall Bierlow Guardians invited local architects to submit competitive designs for temporary hospital accommodation for twenty-four beds. The design submitted by Mr. Arthur Kenyon, A.R.I.B.A., of London and Sheffield, has been selected.

Skelton.—The Skelton and Brotton Urban Council has decided to build an infectious diseases hospital.

Sunderland.—For alterations to the isolation hospital at Ford and the erection of a tuberculosis pavilion a tender of £7,978 has been accepted.

Warrington.—The Guardians have appointed a special committee to consider a scheme for enlarging the imbecile and epileptic accommodation at a cost of £12,000.

Watford.—Mr. W. H. Syme, F.R.I.B.A., of Vicarage Road, Watford, is architect for additions at the Guardians' premises. Tenders are under consideration.

Windsor.—A tender of close on £10,000 has been accepted by H.M. War Office for a new hospital at Combermere Barracks.

### Appointments.

Dr. Thomas Holmes, M.D., M.S. (Lond.), has been appointed senior surgeon on the honorary staff of the Cheltenham General Hospital, in succession to Mr. W. R. Buckell, who has resigned. There were four candidates for the post, one being a woman. Dr. Holmes studied at Guy's, where he was Second Exhibitioner three years running, passed the Intermediate M.B. with honours in anatomy, and finally the London University Gold Medal as Master in Surgery.

### Gifts and Bequests.

Dr. Barnardo's Homes have received £10 10s. each from the Skinners' Company, the Tallow Chandlers' Company, and the West London Police Court.

The Royal Sea Bathing Hospital for Surgical Tuberculosis, Margate, has received a further sum of £200 from "A Friend to Children" to complete the endowment of a bed.

Mrs. Jane Bagshawe, who left estate valued at £5,844 gross, has bequeathed, subject to two life interests, £1,000 to the Leeds Infirmary and £250 to the said infirmary for consumptive patients.

Mr. Edward Herbert Bayldon, of Dawlish, who left estate provisionally valued for probate at £100,000, has bequeathed £200 to the Devon and Exeter Hospital, and £100 to the Dawlish Cottage Hospital.

The Royal Free Hospital, Gray's Inn Road, W.C., has received twenty-five guineas from Lloyds Bank (Limited), Holborn Circus Branch, for the new out-patient department, and £5 5s. from the Patent Steam Carpet Beating Company for the same purpose.

Manchester Children's Hospital, Pendlebury, has received the following legacies during the past month:—£90 from the late Mr. Julius Steinberg, £504 from the late Miss Helen Swindells, being a share of her residuary estate, also a donation of £200 from Mr. Robert G. Peel.

In response to a recent appeal by the Lady Mayoress, as president, and Lady Mackinnon, as chairman, of the City of London Branch of the British Red Cross Society, Lady Perks has contributed £25, the Clothworkers' Company £20, Messrs. Hope, Pollock and Co. £10 10s., and Messrs. Myers and Co. £10 10s.

Miss Margaret Beatrice Hudson, of Bushey Heath, has left £500 each to the Leeds General Infirmary and King Edward's Hospital Fund, and £200 each to the Bushey Heath Cottage Hospital, the Royal National Hospital for Consumption and Diseases of the Chest, Ventnor, and the Royal Hospital for Incurables, Putney.

The Tallow Chandlers' Company have granted to the London Hospital, £21; East London Hospital for Children, £15 15s.; Merchant Seamen's Orphan Asylum, Middlesex Hospital, Royal Waterloo Hospital for Children and Women, Guy's Hospital, Royal Hospital for Incurables, Putney, Great Northern Central Hospital, St. Bartholomew's Hospital, St. Peter's Hospital for Stone, etc., and Dr. Barnardo's Homes each £10 10s.; to the West Ham and Eastern General Hospital and the Surgical Aid Society each £5 5s.

A DOCTOR'S BEQUEST.

Dr. Frederic Bagshawe, J.P., M.D., F.R.C.P., Mayor of St. Leonards, 1897-8, a former President of the South-East Branch of the British Medical Association, has left estate valued at £80,817 gross, with net personalty £78,534. He left £1,000 to the East Sussex Hospital, and 20 £50 shares in the Hastings Cottage Improvement Society, directing that these shares shall not be disposed of for ten years after his death.

Mr. Thomas Manners Smith, M.A., M.B., B.C., M.R.C.S., demonstrator in anatomy at Cambridge University, who died intestate and a bachelor, has left estate valued at £1,215 gross.

Mr. Edward Blakeway I'Anson, M.A., F.R.I.B.A., Surveyor to St. Bartholomew's Hospital, and a Vice-President of the Surveyors' Institution, has left estate of the gross value of £87,194, of which £70,746 is net personalty.

### Obituary.

Dr. John Thomas Roberts, B.M., died suddenly on Christmas Eve at the early age of forty-one. He was educated at the University College of Wales, Aberystwyth, and at Guy's Hospital, and graduated B.M. of London. He was afterwards in practice at Chester.

### Doctors' Wills.

Dr. John James Charles, M.D., D.Sc., late demonstrator of and lecturer in anatomy at Queen's College, Belfast, left personal estate in the United Kingdom valued at £41,017, of which £13,856 is in England.

Messrs. W. and J. Burrow, of The Springs, Malvern, have received a Royal Warrant of Appointment as Purveyors of the "Alpha Brand" Malvern Water to the King—a tribute, the public will note, to the value of springs in this country.

HONOUR FOR RONUK.

The proprietors of Ronuk Sanitary Polish have had the honour to receive a Royal Warrant of Appointment to Her Majesty Queen Alexandra.

